# SARS-CoV-2 Infection in Healthcare Professionals and General Population During “First Wave” of COVID-19 Pandemic: A Cross-Sectional Study Conducted in Sicily, Italy

**DOI:** 10.3389/fpubh.2021.644008

**Published:** 2021-05-13

**Authors:** Claudio Costantino, Emanuele Cannizzaro, Maria Gabriella Verso, Fabio Tramuto, Carmelo Massimo Maida, Guido Lacca, Davide Alba, Livia Cimino, Arianna Conforto, Luigi Cirrincione, Giorgio Graziano, Sara Palmeri, Stefano Pizzo, Vincenzo Restivo, Alessandra Casuccio, Francesco Vitale, Walter Mazzucco

**Affiliations:** ^1^Department of Health Promotion, Maternal and Infant Care, Internal Medicine and Medical Specialties (PROMISE) “G. D'Alessandro,” University of Palermo, Palermo, Italy; ^2^COVID-19 Surveillance Western Sicily Reference Laboratory, Palermo University Hospital, Palermo, Italy

**Keywords:** SARS-CoV-2 infection, COVID-19, healthcare workers, personal protective equipment, traffic control building, influenza vaccination, psycho-physical support

## Abstract

On December 31, 2019, an outbreak of lower respiratory infections was documented in Wuhan caused by the severe acute respiratory syndrome coronavirus 2 (SARS-CoV-2). Since the beginning, SARS-CoV-2 has caused many infections among healthcare workers (HCWs) worldwide. Aims of this study were: a. to compare the distribution among the HCWs and the general population of SARS-CoV-2 infections in Western Sicily and Italy; b. to describe the characteristics of HCWs infected with SARS-CoV-2 in the western Sicilian healthcare context during the first wave of the epidemic diffusion in Italy. Incidence and mean age of HCWs infected with SARS-CoV-2 were comparable in Western Sicily and in the whole Italian country. The 97.6% of infections occurred in HCWs operating in non-coronavirus disease 2019 (COVID-19) working environments, while an equal distribution of cases between hospital and primary care services context was documented. Nurses and healthcare assistants, followed by physicians, were the categories more frequently infected by SARS-CoV-2. The present study suggests that healthcare workers are easily infected compared to the general population but that often infection could equally occur in hospital and non-hospital settings. Safety of HCWs in counteracting the COVID-19 pandemic must be strengthened in hospital [adequate provision of personal protective equipment (PPE), optimization of human resources, implementation of closed and independent groups of HCWs, creation of traffic control building and dedicated areas in every healthcare context] and non-hospital settings (influenza vaccination, adequate psychophysical support, including refreshments during working shifts, adequate rest, and family support).

## Introduction

On December 31, 2019, an outbreak of unexplained lower respiratory infections was documented in Wuhan, the largest metropolitan area in China's Hubei Province, and reported for the first time to the WHO National Office in China (1). The etiology of this disease was attributed to a new virus belonging to the family of coronaviruses (CoVs), renamed severe acute respiratory syndrome coronavirus 2 (SARS-CoV-2) (2).

Previous outbreaks of CoV have been documented in the past, such as severe acute respiratory syndrome renamed coronavirus (SARS-CoV-1) in 2002/2003 and Middle East respiratory syndrome coronavirus (MERS-CoV) in 2012 (3).

In early March 2020, the new virus demonstrated high contagiousness, and its worldwide spread has reached the epidemiological criteria necessary to be declared a pandemic (4). To date, about 73 million cases of SARS-CoV-2 infection and more than 1.6 million deaths due to coronavirus disease 2019 (COVID-19) have been observed worldwide (4).

In the last months, in Europe, there has been an epidemiological transition of the SARS-CoV-2 epidemic, with a sharp drop to 43 years in the average age of the population contracting the infection (5, 6).

Virus circulation occurs more frequently in younger age groups due to a greater reopening of commercial activities (including meeting places) and greater mobility, and this variation in transmission dynamics (with the onset of cases and outbreaks linked to recreational activities) was recently confirmed by epidemiological data supporting the evidence of the occurrence of a possible second wave of the pandemic (6).

SARS-CoV-2 has also caused many infections among healthcare workers (HCWs) worldwide (7). The Chinese National Health Commission stated that more than 3,300 health workers nationwide had been infected, and many of them died during the first epidemic wave that occurred in China (8).

HCWs are particularly vulnerable compared to the general population, as demonstrated by the continuous increase in cases, because the coronavirus is highly contagious and members of medical staff are increasingly exposed to viral particles, while often not being provided with adequate protective equipment (9). In addition, a combination of stress, long working hours, and night work could make their immune system more vulnerable than normal (10–13).

In addition, the Commission Directive (EU) 2020/739 included SARS-CoV-2 in the list of biological agents known to infect humans and amending Commission Directive (EU) 2019/1833 (14).

In Italy, to date, more than 80,000 HCWs have been infected with SARS-CoV-2 and hundreds have died due to COVID-19 (6).

Aims of this observational study were to compare the distribution among the HCWs and the general population of SARS-CoV-2 infection cases that occurred in Western Sicily and Italy and to describe the main characteristics of HCWs infected with SARS-CoV-2 in the western Sicilian healthcare context during the first wave of the SARS-CoV-2 epidemic diffusion in Italy. This analysis could contribute to investigate any differences of SARS-CoV-2 infection incidence according to health care setting, professional role, or working environment.

## Materials and Methods

A cross-sectional study was conducted in Western Sicily, to date, a COVID-19 low-incidence area accounting for about 47.5% of the total Sicilian resident population (4,968,410 inhabitants), to highlight the characteristics of HCWs infected with SARS-CoV-2 during the period between February 26, 2020 (first laboratory-confirmed case of SARS-CoV-2 infection diagnosed in Sicily among the general population), and May 3, 2020 (the last days of the “lockdown” that began in Italy on March 8, 2020).

Data were obtained from the COVID-19 surveillance systems in place at the local health facilities (LHFs) of four western Sicilian provinces: Agrigento (429,611 inhabitants), Caltanissetta (260,779 inhabitants), Palermo (1,243,328 inhabitants), and Trapani (428,337 inhabitants).

To this end, a dedicated electronic format has been structured and then shared with officials from the prevention departments of recalled LHFs, identified to collect data on HCWs who resulted positive to SARS-CoV-2.

The variables collected were gender, age (mean, standard deviation), period of SARS-CoV-2 infection diagnosis (I subperiod = from February 26, 2020, to March 31, 2020; II subperiod = from April 1, 2020, to May 3, 2020), province (Agrigento, Caltanissetta, Palermo, and Trapani), healthcare context (hospital, local health structure, or primary care service), professional role (physician, nurse, healthcare assistant, laboratory technician), working environment (COVID-19 or not COVID-19 hospital/ward/primary care service).

The diagnosis of SARS-CoV-2 infection, in accordance with WHO guidelines, was considered for nasopharyngeal swab specimens confirmed at real-time polymerase chain reaction (RT-PCR) by one of the diagnostic laboratories authorized by the Sicilian regional health department or by the Italian Ministry of Health (4, 6).

Lastly, data obtained from the LHFs were compared with data on the general population available from the open-access database provided by the Italian national surveillance system for COVID-19 epidemic and with data on HCWs provided by the Italian National Health Institute (6).

The study was approved by the Ethical Committee Palermo 1 of the University Hospital of Palermo (session n. 7 of July 13, 2020).

### Statistical Analysis

Quantitative variables were normally distributed and summarized as means with their standard deviations (±SD), while absolute and relative frequencies were calculated for qualitative variables. Chi-square test was performed to compare the distribution of SARS-CoV-2-positive cases. Statistical significance was set with a *p* < 0.05.

All data were entered into an electronic database created by Excel 16.0 software. Descriptive statistics were performed using EpiInfo® ver. 3.5.1 software.

## Results

All SARS-CoV-2 infection cases among HCWs in Western Sicily registered by the Sicilian Health Department were contacted and reported in the analysis.

In Table 1, the comparison between Western Sicily and Italy of the distribution of SARS-CoV-2 infection cases, confirmed by RT-PCR molecular test, among HCWs and the general population is represented, which occurred during the first wave of the COVID-19 pandemic.

**Table 1:** Distribution of laboratory-confirmed diagnosis of SARS-CoV-2 infections among HCWs and the general population.

**Location**	**SARS-CoV-2 laboratory-confirmed infection**			
	**Overall**	**HCWs**	**General**	***p*-value**
			**population**	
	***n*** **(%)**			
Western sicily^*^	944 (100)	85 (9.0)	859 (91.0)	0.18
Remaining part of Italy^*^	209,773 (100)	21,795 (11.6)	187,978 (88.4)	
Italy overall	210,717 (100)	21,880 (10.4)	188,837 (89.6)	

* *Comparison between Western Sicily and Italy (February 26, 2020, to May 3, 2020).*

*HCWs, healthcare workers; SARS-CoV-2, severe acute respiratory syndrome coronavirus 2*.

In Western Sicily, during the period in this study, 944 SARS-CoV-2-positive subjects were documented overall. Of these, 85 (9%) were HCWs and the remaining 859 (91%) occurred in the general population.

The total number of SARS-CoV-2-positive subjects reported in the remaining part of Italy in the same period was of 209,773, with 21,795 (11.6%) cases occurring in HCWs and 187,978 (88.4%) documented in the general population.

However, no statistical difference was reported when comparing the distribution of SARS-CoV-2-positive cases between Western Sicily and the remaining part of Italy (*p*-value 0.18).

Table 2 summarizes the characteristics of the 85 HCWs working in the Western Sicily healthcare context with a laboratory-confirmed diagnosis of SARS-CoV-2 infection reported during the first pandemic. Among them, 52 (61.2%) were males and 33 (38.8%) were females, with an average age of 46.4 (SD ± 10.8) years. The majority of HCWs (61; 71.8%) were infected in the first subperiod of the study (from February 26, 2020, to March 31, 2020), while the remaining part (24; 28.2%) during the second subperiod surveyed (from April 1, 2020, to May 3, 2020).

**Table 2:** Characteristics of 85 HCWs with a laboratory-confirmed diagnosis of SARS-CoV-2 infection working in the western Sicilian healthcare context (February 26, 2020, to May 3, 2020).

**Characteristics**		***n***	**(%)**
Gender	Male	52	61.2
	Female	33	38.8
Period of diagnosis	I subperiod in study^*^	61	71.8
	II subperiod in study^**^	24	28.2
Age	Mean (± SD)	46.4	± 10.8
Province	Agrigento	21	24.7
	Caltanissetta	9	10.6
	Palermo	35	41.2
	Trapani	20	23.5
Healthcare context	Hospital	43	50.6
	Local health structure or primary care service	42	49.4
Professional role	Physician	22	26.8
	Nurse	29	34.1
	Healthcare assistant	29	34.1
	Laboratory technician	5	4.9
Working environment (hospital/ward/primary care service)	COVID-19	2	2.4
	Non-COVID-19	83	97.6

*COVID-19, coronavirus disease 2019; HCWs, healthcare workers; SARS-CoV-2, severe acute respiratory syndrome coronavirus 2; SD, standard deviation.*

* *From February 26, 2020, to March 31, 2020;*

** *from April 1, 2020, to May 3, 2020*.

The cases were distributed in the four LHFs as follows: 21 (24.7%) Agrigento, nine (10.6%) Caltanissetta, 35 (41.2%) Palermo, 20 (23.5%) Trapani.

Among the laboratory-confirmed diagnosis of SARS-CoV-2 infection occurring in HCWs from the western Sicilian healthcare context, 43 (50.6%) worked in a hospital and 42 (49.4%) in the primary care services or in other structures belonging to the LHFs.

With regard to the working environment, two (2.4%) HCWs operated in a COVID-19 hospital/ward or primary care service considered at high risk of exposure to SARS-CoV-2, while 83 (97.6%) of the SARS-CoV-2-infected HCWs were from a non-COVID-19 working context.

Lastly, of the 85 SARS-CoV-2-positive HCWs, 22 (26.8%) were physicians, 29 (34.1%) were nurses, five (4.9%) were laboratory technicians, and 29 (34.1%) were healthcare assistants.

## Discussion

Since the end of February 2020, when the first evidence of local transmission of SARS-CoV-2 was observed in Italy, many HCWs have also been infected by the pandemic virus (6).

We conducted a cross-sectional study to compare the distribution among the HCWs and the general population of SARS-CoV-2 infection cases occurring in Western Sicily and Italy and to further document the main characteristics of the SARS-CoV-2-infected HCWs operating in the western Sicilian healthcare system, laying in an Italian COVID-19 low-incidence context, during the first wave of the SARS-CoV-2 pandemic. To this end, we accessed data from the COVID-19 surveillance systems in place at the Sicilian LHFs and the open-access database provided by the Italian national surveillance system for COVID-19 epidemic.

During the first wave of the pandemic, the distribution of SARS-CoV-2-positive cases among HCWs and the general population was comparable in Western Sicily and in the whole Italian country.

This finding related to the proportion of SARS-CoV-2-infected HCWs on the overall number of SARS-CoV-2-positive cases should be further analyzed according to the different incidences of COVID-19 cases decreasing gradient from north to south reported in Italy (6). The greatest burden of the disease documented for northern Italian regions could be attributable to several factors such as the delay in applying the lockdown, the import–export with China, the influenza vaccination coverage rates, the mean seasonal temperatures, and the different impacts of air pollution among different Italian regions, and the role of these factors should be considered when investigating the impact of COVID-19 on HCWs as well (6, 15).

Of interest, the risk of contracting COVID-19 among HCWs is one of the highest because regardless of the specific exposure related to healthcare settings, outside working hours, they are also exposed to the same risk and pathways of contagion reported in the community (16–18).

More in depth, HCWs resulted at very high risk of contracting SARS-CoV-2 infection because of several factors: the healthcare setting (not necessarily an at-risk ward that required tight preventive measures), longer working hours, night shifts that adversely affect the normal waking–sleep rhythm, and poor hand hygiene following contact with patients potentially affected by COVID-19 or the inadequacy of the personal protective equipment (PPE) and of the training in its correct use, provided by the healthcare organization during a pandemic (17, 18).

Our study documented that HCWs at higher risk of SARS-CoV-2 infection during the first pandemic wave were males and had an average age of 46.6 years. These findings are in line with the ones reported in Italy overall during the same time frame (6).

Of relevance, the majority of COVID-19 cases occurring in western Sicilian HCWs were reported during the first month of the first epidemic wave, probably reflecting an improvement over time of the prevention and control measures implemented in the healthcare setting. During the COVID-19 pandemic, HCWs represent the most valuable resource in each country worldwide (19, 20). However, knowledge about the effectiveness of PPE on HCWs caring for patients infected with the novel coronavirus is continuously increasing (21).

### Practical Implication

A recent clinical case described the clinical outcome of 41 HCWs who were exposed to aerosol-generating procedures for at least 10 min at a distance of <2 m from a patient with severe pneumonia before the laboratory-confirmed diagnosis of COVID-19 was known (22). Despite the fact that 85% of HCWs were exposed during a procedure involving aerosol generation by the patient while wearing a surgical mask or N95 mask, none of them developed symptoms and all molecular tests resulted negative (22).

These pieces of evidence suggest that surgical masks, hand hygiene, and other standard procedures are effective in protecting HCWs from the SARS-CoV-2 infection. A single case report is not sufficient to determine the best way to protect HCWs from COVID-19, but it is sufficient to suggest that further studies are needed (23).

Moreover, with the present study, we tried to assess the possible increased vulnerability of HCWs to develop the SARS-CoV-2 infection according to their healthcare context and professional role. In particular, the number of HCWs with a confirmed laboratory diagnosis of SARS-CoV-2 working in hospitals was comparable to the one documented for HCWs operating in the primary care services or in other structures belonging to the LHFs, suggesting a similar level of risk for contracting the disease (23, 24). A possible interpretation of these data can be related to a contagion in the LHFs, especially among HCWs working in long-term care facilities for elderly and disabled persons (25).

Therefore, HCWs operating at the territorial services or in a non-COVID-19 setting, on one side, and the ones working at the hospital wards (not dedicated to the treatment of patients affected by COVID-19), could not have greater protection from the virus than their colleagues working in COVID-19 units (26).

In accordance with this hypothesis, 97.6% of the HCWs infected by SARS-CoV-2 in western Sicilian healthcare settings worked in environments irresolute to COVID-19 patterns of care. In this perspective, the experiences conducted in low-resource countries in the management of epidemics within outpatient settings while having a reduced number of available health personnel could be of interest (27–29).

However, one of the most important reasons for early infection among general ward medical staff is the frequent misconduct of admitting patients to the ward without implementing preventive and protective measures (30).

On the contrary, infection rates in the most protected intensive care and emergency wards were lower in unannounced cases of illness (31).

Recent research published in the *Journal of the American Medical Association* found that of the 138 patients studied in a hospital in Wuhan, 29% were HCWs, of whom 31 (77.5%) were working in general wards, seven (17.5%) in emergency rooms, and two (5%) in intensive care units (32).

Of interest, it was assumed that more than 10 HCWs in this ward were infected by a patient with abdominal symptoms and admitted to the surgical ward (32).

One of the reasons for the difficulties in diagnosing COVID-19 and the consequent placement of the patient in wards without adequate measures to contain the virus was found in the atypical presentation symptoms in the initial phase in some patients infected with SARS-CoV-2, such as the gastrointestinal symptoms of the patient under examination, which probably contributed to the rapid spread of the infection among HCWs (32).

Of interest, Traffic Control Bundling (TCB) tool was proven to be effective in drastically reducing infection rates among HCWs in Taiwan during the SARS epidemic (33). Furthermore, the greatest susceptibility to respiratory infectious diseases, such as SARS, was documented for HCWs (24); moreover, data from seven hospitals in China showed an incidence of up to 13.53% of SARS-infected HCWs in intensive care (intensive care units) (26).

More in depth, TCB approach requires the identification of (1) a pre-triage outside hospital (in tents or other shelters), ensuring that patients are triaged in outdoor screening stations so that sick patients are directed to a contamination zone; and (2) risk zones, clearly delimiting separate zones, including a contamination, transition, and clean zone, each separated by control points. Therefore, it has been suggested to implement the TCB model to face manage the COVID-19 outbreak (33).

HCWs should be adequately trained on TCB protocols including good dressing and undressing practices, proper use of all appropriate personal safety equipment (e.g., respirators and eye protection), and how to move safely between areas (34).

Last but not least, some differences between the working categories were observed in the present study. Nurses and healthcare assistants were the categories most affected by the pandemic virus as compared to physicians probably because their role involves greater physical proximity to the patient (dressing, cleaning, feeding) and, therefore, a greater chance of viral transmission, differently from laboratory technicians who manipulate the swabs in a controlled laboratory (35).

Generally, HCWs could also be easily infected outside work environment in the familiar context. The lack or incorrect use of preventive measures in order to prevent the transmission of SARS-CoV-2 infection is usually more frequent among family members or friends (36).

The strong recommendation of influenza vaccination should be of paramount importance during the 2020/2021 influenza season for certain categories such as HCWs, medical and healthcare residents/students/trainees, police officers, fire workers, and other workers of public utility (37).

In that direction, three Italian regions have tried to introduce local policies or laws that mandate influenza vaccination among HCWs, considering them at high risk for contracting and spreading influenza viruses that could contribute to the burden and overcrowding of health care systems together with SARS-CoV-2 during the next cold season (38, 39).

Could be also useful is the possibility for some HCWs, directly involved in the care of COVID-19-infected patients, to separate temporarily from their families in dedicated COVID-19 hotels for HCWs in order to protect their parents from the risk of secondary infection (40, 41).

Also the mental health and the COVID-19-related mental health effects in the workplace of frontline health and social care professionals should be taken into account in the preventive measures to be considered in the future (42, 43). For instance, HCWs infected by SARS-CoV-2 expected to experience higher levels of stigma among colleagues and in the working setting, reporting increased psychological distress (44–46).

### Limitations and Strengths

The main limitation of the present study is linked to its purely descriptive design and to the small number of cases collected that could not be representative of the national context where a different epidemiologic impact was observed, with a COVID-19 incidence decreasing from north to south.

Moreover, further analytical studies, such as ones with a case-control design, should be conducted in the near future to evaluate the different risk factors associated with the infection among HCWs who tested positive for SARS-CoV-2.

Also for future research on SARS-CoV-2 infection of HCWs, an in-depth analysis of psychological variables should be considered.

Finally, the present study contributes to the research conducted on SARS-CoV-2 infections among HCWs in other countries. Specifically, the main elements of originality of the present research can be summarized as follows:

1. HCWs, independently if working in COVID-19 or non-COVID-19 environment, are easily infected in geographical areas with a higher incidence of SARS-CoV-2 infection (Northern vs. Southern Italy during the “first-wave” interesting Italy) (6).
2. There are no significant differences in SARS-CoV-2 infection incidence in accordance to working environment or healthcare context, suggesting a higher risk of transmission among HCWs in non-healthcare settings, where preventive measures are usually neglected.

## Conclusions

In conclusion, it is essential to highlight how the safety of HCWs must be strengthened at a global level through certain essential preventive measures (23).

During the next phases of the COVID-19 pandemic, some preventive measures in order to reduce the SARS-CoV-2 infections among HCWs should be strongly encouraged.

Adequately providing suitable PPE, optimizing human resources in the different healthcare settings, implementing closed and independent groups of HCWs that can be easily isolated, creating traffic control building and dedicated areas for suspected and confirmed cases of SARS-CoV-2 infection, offering influenza vaccination to all HCWs, and providing everything that can guarantee adequate psychophysical support (including refreshments during working shifts, guaranteeing adequate rest, and family support) are among the most important strategies to be considered.

Finally, it should be considered that HCWs are easily infected compared to the general population, but often that infection could occur in hospital and non-hospital settings.

Preventive measures in order to contrast SARS-CoV-2 infection should be continuously applied by the general population until the end of this pandemic, also contributing to a reduction of HCW infections (36).

## Data Availability Statement

The raw data supporting the conclusions of this article will be made available by the authors, without undue reservation.

## Ethics Statement

The studies involving human participants were reviewed and approved by Ethical Committee Palermo 1 of the University Hospital of Palermo (session n.7 of July 13, 2020). Written informed consent for participation was not required for this study in accordance with the national legislation and the institutional requirements.

## Author Contributions

CC and WM were responsible for the study conception, methodology, and writing the original draft of the manuscript. EC and FV were responsible for reviewing and editing the manuscript and supervision of the project. ACa, VR, GL, and MV were responsible for the statistical analysis. CM and FT were responsible for data curation. LCir, GG, SPa, and SPi were responsible for data collection. DA, ACo, and LCim were responsible for the investigation in the western Sicilian local health facilities. All authors contributed to the article and approved the submitted version.

## Conflict of Interest

The authors declare that the research was conducted in the absence of any commercial or financial relationships that could be construed as a potential conflict of interest.
